# High Oct4 expression: implications in the pathogenesis of neuroblastic tumours

**DOI:** 10.1186/s12885-018-5219-3

**Published:** 2019-01-03

**Authors:** Ezequiel Monferrer, Rebeca Burgos-Panadero, Maite Blanquer-Maceiras, Adela Cañete, Samuel Navarro, Rosa Noguera

**Affiliations:** 1 Pathology Department, Medical School, University of Valencia-INCLIVA, Av. Blasco Ibáñez, 15, 46010 Valencia, Spain; 2 CIBERONC, Madrid, Spain; 3 Pediatric Oncology Unit, University and Polytechnic Hospital La Fe, Valencia, Spain

**Keywords:** Oct4, Digital image analysis, Neuroblastic tumours

## Abstract

**Background:**

Neuroblastic tumours (NBTs) are paediatric solid tumours derived from embryonic neural crest cells which harbour their own cancer stem cells (CSC). There is evidence indicating that CSC may be responsible for tumour progression, chemotherapy resistance and recurrence in NBTs. Oct4 is a transcription factor which plays a key role in mammal embryonic development and stem cell fate regulation. The aim of the study is to elucidate the clinical significance of Oct4 in NBTs.

**Methods:**

We studied Oct4 expression in 563 primary NBTs using digital image quantification. Chi-square test was applied to analyse the correlation between histopathology and the Oct4^+^ cell percentage. Survival analysis was carried out with Kaplan-Meier curves and log-rank test. Additionally, a multivariate Cox regression analysis with the stepwise backwards (Wald) method was undertaken to calculate the impact of Oct4 expression level on survival.

**Results:**

We found that tumours with a high proportion of cells expressing Oct4 correlated statistically with undifferentiated and poorly differentiated neuroblastoma / nodular ganglioneuroblastoma, and that Oct4 expression was not present in ganglioneuroma (*p* < 0.05). Statistical analysis also indicated a relationship between high Oct4 expression levels, high-risk patients according to the International Neuroblastoma Risk Group pre-treatment classification parameters, larger blood vessels and low survival rates.

**Conclusions:**

These results suggest that the Oct4 gene may regulate NBT pathogenic differentiation pathways, and should thus be considered as a target for knockdown when developing novel therapies for high-risk NBT patients.

## Background

Neuroblastic tumours (NBTs) are the most common paediatric extracranial solid tumours, responsible for 15% of childhood cancer-related deaths [1, 2]. They arise from the sympathetic nervous system and are characterized by being highly clinically and genetically heterogeneous tumours [3–5]. Despite the advances made in treatment and risk classification of NBTs [6–8], the basic molecular pathways of NBT pathogenesis remain unclear.

Different stem cell-related genes have been studied in cancer, following the cancer stem cell (CSC) hypothesis [9, 10]. This supposition presumes that stem cell-related genes expressed in CSCs may be responsible for relapse and treatment failure, becoming invasive and potentially immortal-like preimplantation embryonic cells [11]. Oct4 is one of those stem cell-related genes, and plays an important role in mammal embryonic development, cellular fate determination and pluripotency maintenance [12–14]. The expression of this transcription factor has been used as a reliable biomarker for germ cell tumours [15, 16] and different somatic cancers [17–19] and has been related to metastasis, aggressiveness and poor prognosis in most of them [20–22].

Previous studies have described Oct4 expression in different human neuroblastic cell lines and side-population cells in NBTs, linking its presence with the stem cell-like phenotype in these tumours [23–25]. The characteristic phenotype implies a stem cell self-renewal capacity, but also the presence of tumour cells with multipotential differentiation properties that can be induced to differentiate in neuroblastic or glial phenotypes. Moreover, Oct4 seems to be linked with NBT neovascularization when co-expressed with Tenascin C in perivascular neuroblastic cells [26], and other studies have reported that Oct4 is also associated with *MYCN* amplification and stages 3 and 4 of the International Neuroblastoma Staging System [27–29]. These studies also suggested that Oct4 could be related to undifferentiated and poorly differentiated neuroblastomas (NB), but no statistically significant correlation was found [27].

Although Oct4 is generally accepted to be correlated with bad prognosis and low survival in cancer, this transcription factor is a good prognostic factor in some carcinomas like testicular germ cell tumours [21]. It also has been postulated that different levels of Oct4 may induce different cell fates due to the multiple interactions of this biomarker, or its isoforms and pseudogenes, with different factors [14, 21]. Therefore, due to the biological complexity of Oct4, the low number of NBT series studied and the differences between methodologies employed, Oct4 expression pattern and its clinical significance in NBTs remain unclear.

As pathology enters the era of personalized medicine, digital pathology has emerged, favouring the adoption of digital image analysis, especially in tissue biomarker research [30]. Computational approaches overcome the limitations of pathologist analysis by producing continuous variable data and providing automation and reproducible analysis, although pathologist validation is required to ensure the accuracy of the image analysis algorithms [31, 32].

In this study, we applied automatic image analysis quantification after an immunohistochemistry analysis approach to assess Oct4 expression in a large cohort of primary NBT samples, and correlated their expression to the International Neuroblastoma Risk Group (INRG) features that have known prognostic value [7]. We also tested if there was a relationship between Oct4 expression and the size and shape of blood vessels, which are morphometric variables related to high-risk NB according to a previous study [33]. In order to determine the prognostic value of Oct4, we assessed the distribution of the cases that presented low or high Oct4-positive (Oct4^+^) cell percentage in each clinicopathological group. The Oct4^+^ cell percentage was the proportion of Oct4^+^ cells in each tumour. The results of these associations provide information about the usefulness of Oct4 biomarkers in pre-treatment risk classification and development of new therapeutic strategies.

## Methods

### Samples and patients

We analysed 563 primary NBTs, including two or more representative cylinders of 1 mm from each tumour, in a total of 33 tissue microarrays (TMAs). Patient samples were referred to the Spanish Reference Centre for Neuroblastoma Biological and Pathological studies (Department of Pathology, University of Valencia-INCLIVA) between 1996 and 2016. Patients were clinically characterized by the paediatric oncologists in charge and by the clinicians of the Reference Centre for NB clinical studies, and were classified according to the INRG pre-treatment stratification criteria [7]. All patients, their relatives or their legal guardians signed the appropriate written informed consent. The present study was approved by INCLIVA’s Clinical Research Ethics Committee (ref. B.0000339).

### Immunohistochemical analysis

Immunohistochemical (IHC) analysis was carried out to detect Oct4 expression in neuroblastic samples. Paraffin-embedded TMAs were cut into 3 μm sections and automatically IHC stained (Autostainer Link 48; Dako, Glostrup, Denmark) with an anti-Oct4 antibody (Roche). Nuclear brown staining was considered an Oct4^+^ result. Stained samples were independently examined and interpreted by the reference pathologist and a researcher using optical microscopy. Cylinders were individually scored according to their staining intensity (0: negative, 1: low, 2: intermediate, 3: high) and their stained cell proportion (0: < 10%, 1: 10–50%, 2: > 50%). The combination of these two subjective scores was a previously required step for subsequent validation of the results obtained from the objective automated image analysis.

### Image analysis

Stained TMAs were digitalized with the Pannoramic MIDI 3DHistech scanner at 20X magnification and Oct4 expression-related parameters were analysed automatically applying the NuclearQuant module of Pannoramic Viewer software (3DHISTECH). Detected artefacts and folded and/or broken regions were considered uninformative and were excluded from the image analysis. Cylinders with scant material and degraded or lost tissue due to processing were considered non-evaluable and were also excluded from the analysis. The total number of cells and Oct4 individual expression were obtained for each cylinder.

### Statistical methods

Oct4 expression data was obtained as the mean Oct4^+^ cell percentage of the different cylinders in each sample. For statistical purposes, the Oct4^+^ cell percentage was dichotomized according to the median value, and cases were grouped as having low (≤ median value) or high (> median value) Oct4^+^ cell percentage. Low Oct4^+^ cell percentage cases also included Oct4 negative (Oct4^−^) tumours. SPSS version 22 was used to perform the statistical analysis, setting the significance level at 95%. Using the Chi-square test we checked that histopathology correlated with the Oct4^+^ cell percentage established groups (low versus high) in order to elucidate their expression pattern in NBTs. We also evaluated statistical correlation between Oct4^+^ cell percentage groups and variables based on the INRG prognostic categories such as patient age (< 18 versus ≥18 months), tumour stage (localized [L1 & L2] versus metastasis [M & Ms]), histological category (ganglioneuroma [GN] & intermixed ganglioneuroblastoma [GNB] versus NB & nodular GNB), neuroblast differentiation degree (undifferentiated [u] versus poorly differentiated [pd] versus differentiating [d]), presence of numerical or segmental chromosomal aberrations (NCA versus SCA), status of *MYCN* (amplified [MNA] versus non-amplified [MNNA]) and integrity of 11q (non-deleted versus deleted). Blood vessel-related variables (size and shape) were also dichotomized according to the median value, and Oct4^+^ cell percentage correlation analysis was performed with the resulting groups (small versus large and regular versus irregular vessels, respectively). Survival analysis was carried out with Kaplan-Meier curves and log-rank test. A multivariate Cox regression analysis with the stepwise backwards (Wald) method was undertaken to calculate hazard ratios and the impact of Oct4 expression level on survival. For this last purpose, only completely described cases for all variables were considered.

## Results

### Clinicopathological characteristics

All 563 cases presented useful information about Oct4 expression, but some cases remained undefined for other clinicopathological factors (11 for patient age, 23 for tumour stage, 5 for tumour category, 50 for differentiation degree, 111 for chromosomal aberrations, 3 for *MYCN* status and 30 for 11q deletion presence). All the clinicopathological characteristics of the samples are shown in Table 1.

**Table 1:** Statistically significant clinical and biological variables related to Oct4 expression

Factor	Number of cases	Oct4^+^ Cell percentage [N (%)]		*p*-value
		Low	High	
Age, months				
< 18	312	272 (87.2)	40 (12.8)	0.008
≥ 18	240	189 (78.8)	51 (21.3)	
Stage				
L1 & L2	333	287 (86.2)	46 (13.8)	0.012
M & Ms	207	161 (77.8)	46 (22.2)	
Category				
NB & nodular GNB	503	414 (82.3)	89 (17.7)	0.007
GN & intermixed GNB	55	53 (96.4)	2 (3.6)	
Differentiation degree				
Undifferentiated	69	52 (75.4)	17 (24.6)	0.007
Poorly differentiated	345	286 (82.9)	59 (17.1)	
Differentiating	99	92 (92.9)	7 (7.1)	
Chromosomal Aberrations				
Numerical	165	155 (93.3)	10 (6.1)	0.000
Segmental	287	215 (74.9)	72 (25.1)	
*MYCN* status				
Non-amplified	480	415 (86.5)	65 (13.5)	0.000
Amplified	80	54 (67.5)	26 (32.5)	
11q				
Not deleted	427	367 (85.9)	60 (14.1)	0.000
Deleted	106	76 (71.7)	30 (28.3)	
Risk				
Not High Risk	453	388 (85.7)	65 (14.3)	0.009
High Risk	110	83 (75.5)	27 (24.5)	
Blood vessel size				
Small area	230	198 (86.1)	32 (13.9)	0.037
Large area	230	181 (78.7)	49 (21.3)	

Prognostic significance of differential Oct4^+^ cell percentage (low when Oct4^+^ cells ≤8.67% and high when Oct4^+^ cells > 8.67%) and the INRG pre-treatment classification factors in NBTs. (Chi-squared test). The risk parameter represents the combination of the INRG factors indicating whether or not NBTs patients are high-risk patients. The blood vessel size is a morphometric variable previously described as a parameter with prognostic implications in high-risk NB [33]. Statistical significance is shown as the obtained p-values (*p*-value< 0.050). *N* Number of cases, *(%)* Fraction of total cases

### Oct4 expression pattern in NBTs

Some Oct4 cytoplasmic immunoreactivity signals were detectable in NBTs. This can be explained by the presence of OCT4 spliced variants with differential expression patterns [34]. Only nuclear stained cells were considered to be positive; Oct4 expression was observed in 185 of 563 cases (32.86%) and among the Oct4^+^ cases, the Oct4^+^ percentage of cells went from 0.02 to 58.44% with a mean value of 11.5% ± 11.5% and a median value of 8.67%. The median value of Oct4^−^ cells was 2596.

Overall, 102 of the Oct4^+^ tumours (55.14%) had a high number of Oct4^−^ cells (> 2596). According to tumour category and differentiation degree, Oct4 expression was frequently found, with statistical significance (*p* = 0.000) in undifferentiated NB (uNB) [30 of 68 cases (44.12%)] and poorly differentiated NB (pdNB) [129 of 345 cases (37.39%)]. There were 3 nodular poorly differentiated GNB included in the pdNB group. The differentiating NB (dNB) and a nodular differentiating GNB (dGNB) were grouped together and presented in 12 of 44 cases (27.27%) with Oct4^+^ cells. Immunoreactivity for Oct4 was only seen in 3 of 38 (7.89%) intermixed dGNB and none of the 17 GN cases were found to be Oct4^+^. When tumour category and differentiation were compared together with the Oct4^+^ percentage of cells per case instead of Oct4^−^ or Oct4^+^ cases, we corroborated that the link between high Oct4^+^ cell percentage and uNB was statistically significant (*p* = 0.022).

**Fig. 1:**
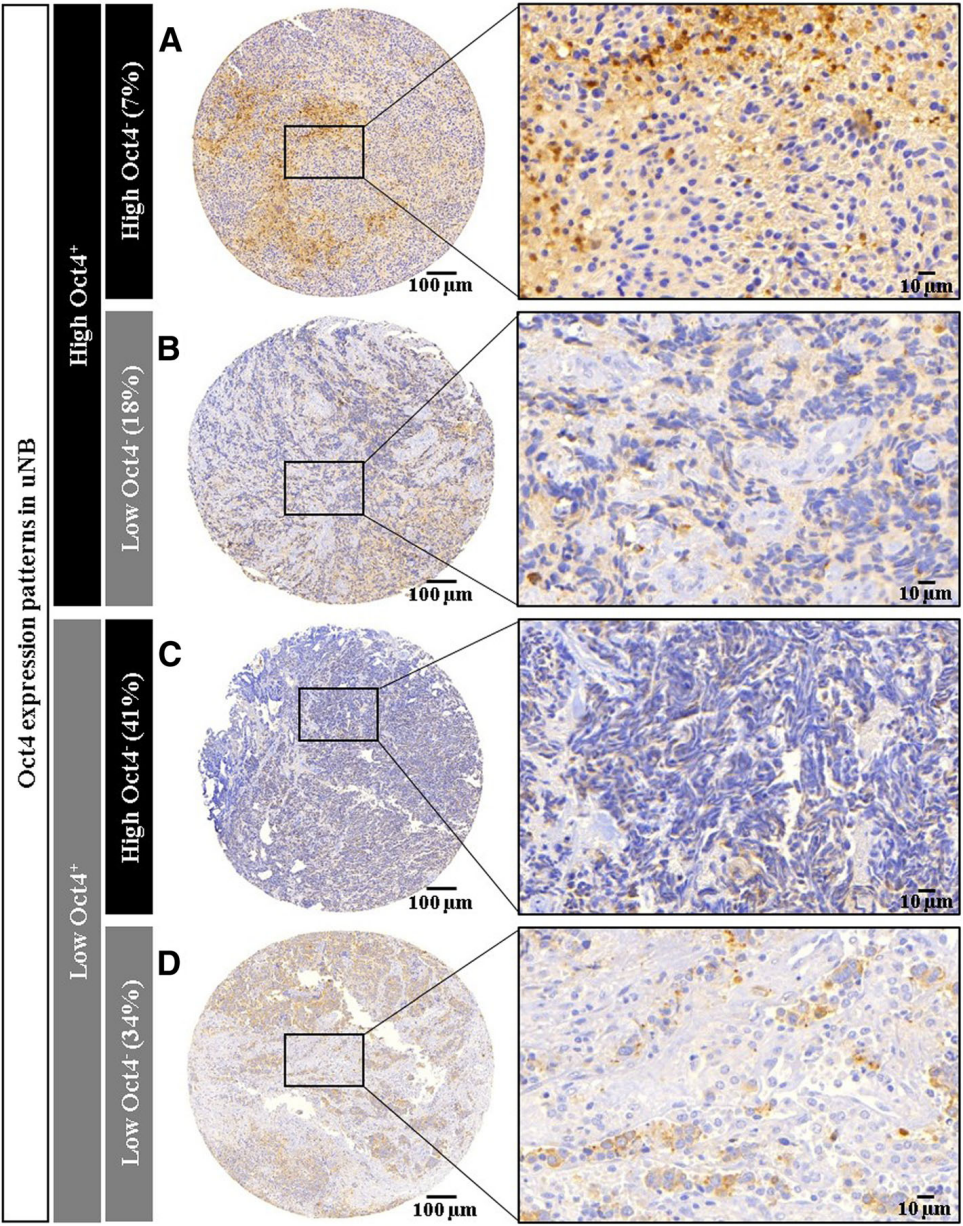
Examples of Oct4^+^ and Oct4^−^ patterns and their frequency in uNB. **a** and **b** Samples corresponding to uNB with high number of Oct4^+^, more than 8.67% of Oct4^+^ cells, **a** intermingle with high number of Oct4^−^ cells (> 2596), **b** with low number of Oct4^−^ cells (≤2596). **c** and **d** Samples corresponding to uNB with low number of Oct4^+^ **c** combine with high number of Oct4^−^ cells, **d** with low number of Oct4^−^ cells

Combining category, tumour differentiation degree and the Oct4^+^ cell percentage, we found that high Oct4^+^ cell percentages were more likely to be found in uNB with a low number of Oct4^−^ cells [12 of 68 cases (17.6%)] rather than in more differentiated tumours or cases with high number of Oct4^−^ cells (Fig. 1). Furthermore, we also observed that cases with low Oct4^+^ cell percentage and low number of Oct4^−^ cells were mainly stroma-rich tumours with a decreasing proportion of Oct4^+^ cells from dNB to intermixed GNB and GN.

### High percentage of Oct4^+^ cells correlates with adverse prognostic factors

Differential expression levels of Oct4 correlated significantly with different clinicopathological factors. High Oct4^+^ cell percentage cases preferentially belonged to high-risk patients according to INRG classification (*p* = 0.009). Studying each prognostic factor independently, we found that the tumours of patients with ≥18 months, metastatic stage, NB or nodular GNB categories, undifferentiated neuroblasts and with presence of SCA, MNA or 11q deletion had a significant relationship (*p* < 0.05) with a greater proportion of high Oct4^+^ cell percentage cases (Table 1).

Additionally, when the relationship between Oct4 expression and blood vessel size and shape was analysed, we found that tumours with high percentages of Oct4^+^ cells significantly presented larger blood vessels (*p* = 0.037).

### High Oct4^+^ expression is related to poor survival

**Fig. 2:**
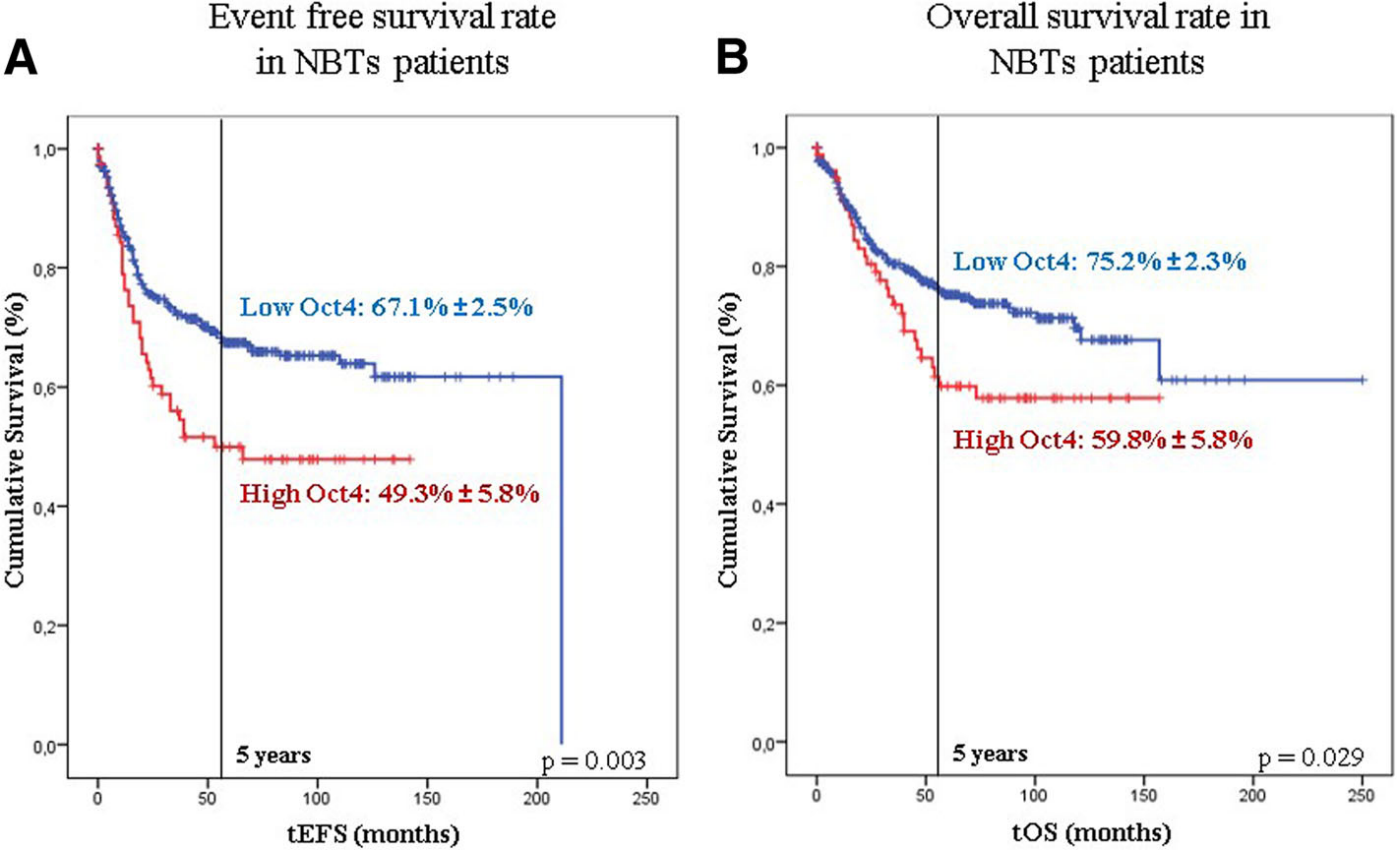
Kaplan-Meier curves representing cumulative EFS (**a**) or OS (**b**) depending on the Oct4^+^ cells proportion. Cases with a low proportion of Oct4^+^ cells present ≤8.67% cells with Oct4 expression and those with high proportion have > 8.67% Oct4^+^ cells. Statistical significance is shown as the obtained *p*-values (*p*-value< 0.050) and survival rates are expressed as percentage of patients without the corresponding event [relapse/death in **a**) or death in **b**)] ± error at 5 years after diagnosis

Kaplan-Meier analysis revealed that high Oct4^+^ cell percentage was significantly related to a decrease in mean survival and cumulative survival at 5 years in both event free survival (EFS) (Fig. 2a; *p* = 0.004) and overall survival (OS) (Fig. 2b; *p* = 0.029). Patients with tumours with low Oct4^+^ cell percentage presented a mean EFS rate of 142.1 months and a mean OS rate of 176.0 months, while those with high Oct4^+^ percentage had a mean EFS rate of 78.1 months and a mean OS rate of 103.3 months. At 5 years, 67.1% ± 2.5% of patients with low Oct4^+^ cell percentage tumours were alive and without relapse, but in patients from the high Oct4^+^ cell percentage group, this value was lower at 49.3% ± 5.8%. If only deceases at 5 years were considered, cumulative OS was 75.2% ± 2.3 and 59.8% ± 5.8% depending on whether the patients had low or high Oct4^+^ cell percentage tumours, respectively. Table 2 shows the relationship between Oct4 expression and different survival-related parameters.

**Table 2:** Relationship between Oct expression and different survival-related parameters

Survival	Oct4 group (N)	Mean Oct4^+^ percentage	Rate of patients with event	Survival mean (months)	Survival at 5 years (%)	*p*-value
EFS	Low (433)	0.62	0.30	141.4 ± 5.3	67.1 ± 2.5	0.003
	High (80)	19.84	0.49	77.1 ± 7.3	49.3 ± 5.8	
OS	Low (433)	0.63	0.23	176.0 ± 8.5	75.2 ± 2.3	0.029
	High (80)	20.32	0.38	103.3 ± 7.6	59.8 ± 5.8	

The events considered in EFS are both relapses and deaths while in OS only deaths are considered events. Rate of patients with event represents the fraction of relapsed or deceased patients of the total. Mean Oct4^+^ cell percentage values correspond to the mean proportion of Oct4^+^ cells presented in each Oct4 tumour group. The mean survival values are represented as the mean survived months without any event occurrence ± error. The 5-year cumulative survival indicates the percentage of patients without event at 5 years after the diagnosis ± error. All these parameters are considered for the Oct4^+^ cell percentage groups in EFS and OS. Statistical significance is shown as the obtained p-values (p-value< 0.050). *N* Total number of patients

In addition, multivariate Cox regression analysis showed that Oct4 could be considered an influential but not determinant variable in OS. This effect may be associated with patient age, tumour stage, *MYCN* status and 11q deletion, which proved to be the most influential variables in OS.

## Discussion

Oct4 participates in different pathways to determine a cell’s fate [14] and its expression has been described and correlated with tumorigenesis, chemotherapy resistance, metastasis, aggressiveness and poor clinical outcomes in medulloblastoma and bladder, gastric, ovarian, lung, colorectal and hepatocellular carcinomas, among others [17–22]. Considering this background, it seems believable that differing numbers of Oct4^+^ cells may have an impact on NBT pathology, and their modulation would serve as a therapeutic approach.

Tumour physics, which highlights the mechanical contribution of microenvironment and cytoskeletal components and their geometry, has been described in cancer progression, metastasis and gene expression [35–38]. Our previous results related to extracellular matrix composition [33, 39–41] and vascular pattern [42] in NBTs also support this fact. For this reason, we suggest that, in accordance with the role of Oct4 in the mammal embryonic development, this biomarker may promote undifferentiated states and cellular proliferation in NBTs, thus increasing tumour aggressiveness through morphological physical stress magnification related to extracellular matrix and cytoskeleton modifications in NB and nodular GNB, similar to the Oct4A role in ovarian cancer [43].

Consistent with this idea and similar to Monajemzadeh et al [27], our data confirmed that u/pdNB and nodular GNB with poorly differentiated or undifferentiated nodules, which are the most unfavourable NBTs according to the INRG classification system [4, 7], are more susceptible to present with a high percentage of Oct4^+^ cells and a low number of Oct4^−^ cells. We also found that Oct4^−^ stroma-rich tumours mainly matched with GN and intermixed GNB, which are the most favourable NBTs [44]. In addition, as Yang et al and Kaneko et al [28, 29] previously indicated, we found that high Oct4^+^ cell percentage cases were significantly correlated with metastatic patterns in NBTs. Other studies also associated Oct4 expression with cell migration boost in different carcinomas [45, 46], which suggests that Oct4 expression may also be involved in metastatic capacity in NBTs by promoting stem cell morphological and growth characteristics in malignant neuroblasts [10].

Oct4 expression also can promote NBT pathogenesis related with angiogenesis. Previous studies reported a correlation between Oct4 expression and vasculogenic mimicry formation in breast cancer [20]. In fact, Oct4^+^ / Tenascin C^+^ perivascular NB cells have also been correlated with neovascularization promotion [26]. In this context Oct4 expression is linked with blood vessel size, which is concordant with our previously defined aggressive vascular pattern [33, 42]. The summative physical effect of Oct4^+^ cells may be at the root of NBT aggressiveness.

The link found between *MYCN* and Oct4 is consistent with Kaneko et al’s [29] results and their model of *MYCN/NCYM*-Oct4 network in *MYCN* amplified human NB, which also explains why high Oct4 expression correlated with poor survival rates among patients with *MYCN* amplification in their analysis. Even though we did not differentiate patients according their *MYCN* status in order to calculate their survival rate independently, our Kaplan-Meier analysis revealed that patients with high Oct4^+^ cell percentage tumours were not only more prone to suffer from tumour relapse, but also had a higher death hazard. Interestingly, we found a statistically significant correlation between high Oct4^+^ cell percentage cases and those harbouring prognostically poor genetic characteristics such as 11q deletion and/or presence of SCA profile.

Moreover, Oct4 downregulation has been strongly associated with cancer invasion suppression [47–49] and chemosensitization [50] in different neoplasms, which indicates that Oct4 could be useful as a therapeutic target.

## Conclusions

In conclusion, high Oct4 expression is preferentially found in undifferentiated NB with a low number of Oct4^−^ cells and correlates with the prognostically poor parameters of the INRG classification. Our results suggest that Oct4 would participate in multiple NBT pathogenic differentiation pathways, including angiogenesis, tumour growth and invasion, but also other physical mechanisms modulated by different factors. These pathogenic pathways could be counteracted by Oct4-based therapies as previously described by Oct4 downregulation. Further to its role in developing novel therapies for high-risk NBT patients, characterization of Oct4 could also help to identify the most susceptible patients with aggressive NBT for these approaches.

## Abbreviations

CSC: Cancer stem cells
d: Differentiating
EFS: Event free survival
GN: Ganglioneuroma
GNB: Ganglioneuroblastoma
IHC: Immunohistochemical
INRG: International Neuroblastoma Risk Group
L1 & L2: Localized tumours
M & Ms: Metastatic tumours
MNA: *MYCN* amplified
MNNA: *MYCN* non-amplified
NB: Neuroblastoma
NBTs: Neuroblastic tumours
NCA: Numerical chromosomal aberrations
OS: Overall survival
pd: Poorly differentiated
SCA: Segmental chromosomal aberrations
TMAs: Tissue microarrays
u: Undifferentiated
